# The Impact of Psychological Stress on Men's Judgements of Female Body Size

**DOI:** 10.1371/journal.pone.0042593

**Published:** 2012-08-08

**Authors:** Viren Swami, Martin J. Tovée

**Affiliations:** 1 Department of Psychology, University of Westminster, London, United Kingdom; 2 Department of Psychology, HELP University College, Kuala Lumpur, Malaysia; 3 Institute of Neuroscience, Newcastle University, Newcastle-upon-Tyne, United Kingdom; The University of South Wales, Australia

## Abstract

**Background:**

Previous work has suggested that the experience of psychological stress may influence physical attractiveness ideals, but most evidence in favour of this hypothesis remains archival. The objective of this study was to experimentally investigate the impact of stress on men's judgements of female body size.

**Methods:**

Men were randomly assigned to either an experimental group, in which they took part in a task that heightened stress (experimental group, *n* = 41) or in which they did not take part in such a task (control group, *n* = 40). Both groups rated the attractiveness of female bodies varying in size from emaciated to obese, completed a measure of appetite sensation, and had their body mass indices (BMIs) measured.

**Results:**

Between-groups analyses showed that the experimental group was matched with the control group in terms of mean age, BMI, and appetite sensation. Further analyses showed that men in the experimental group rated a significantly heavier female body size as maximally attractive than the control group. Men in the experimental group also rated heavier female bodies as more attractive and idealised a wider range of female figures than did the control group.

**Conclusion:**

This study found that the experience of stress was associated with a preference among men for heavier female body sizes. These results indicate that human attractiveness judgements are sensitive to variations in local ecologies and reflect adaptive strategies for dealing with changing environmental conditions.

## Introduction

It is now widely-acknowledged that body size ideals are, in part at least, shaped by an individual's resource security, such that heavier body sizes are preferred where or when resources are unpredictable or unavailable [1]–[2]. This proposition highlights the fact that a primary function of adipose tissue is the storage of calories, which in turn suggests that body fat is a reliable predictor of food availability [3]. In situations marked by resource uncertainty, therefore, individuals should come to idealise heavier individuals [2], as fatness would be associated with access to resources. Conversely, thinness in such contexts may be associated with increased incidence of ill-health [4] and, for women, ovulatory irregularities and lower capacity to support pregnancy [5].

Several lines of evidence support this reasoning. First, cross-cultural studies have reported a strong inverse relationship between socioeconomic status (a covariate of resource security) and ideal body size [4], [6]–[9]. Second, experimental studies have shown that hunger has an effect on men's body size preferences, such that hungry men prefer a significantly heavier body size than satiated men [3], [10]–[12]. These findings mirror reports that hunger intensifies selection for a larger body size in non-human species, such as water-spiders [13]. Nevertheless, it should be noted that the strength of the relationship may depend on the way in which resource security is operationalised: at least one study has failed to replicate the preference for a heavier body size when resource security was experimentally manipulated in terms of financial satisfaction [14].

Related work has also suggested that the experience of stress may affect body size preferences. Specifically, the Environmental Security Hypothesis [15]–[16] suggests that, when socioeconomic or individual conditions are threatening or uncertain, individuals will prefer others with more mature physical characteristics, including a heavier body size, compared to their preferences in non-threatening conditions. This is because physical maturity is associated with the ability to handle threatening situations and because more mature physical features may communicate attributes such as strength, control, and independence during periods when such qualities should be most desired [15]. To date, however, most of the evidence in favour of the Environmental Security Hypothesis is archival in nature: there is evidence, for example that American actresses with more mature facial and bodily features are more popular during periods of socioeconomic hardship [15], [17].

By contrast, experimental tests of the Environmental Security Hypothesis are currently lacking. In one study, Pettijohn and Tesser [18] experimentally manipulated stress by making female and male participants believe they would receive either benign or harmful shocks. These authors reported that, in the stress condition, participants preferred women with decreased eye size, whereas when stress was absent they preferred women with increased eye size. To date, however, experimental studies have not investigated the impact of psychological stress on perceptions of body size, which would appear to be a more direct candidate for assessing the impact of stress on physical attractiveness ideals for a number of reasons. First, although maturity is a broad construct, body size appears to be an important signal of both physical and psychological maturity, such that heavier and taller figures are perceived as more mature and also as having more mature personality traits [17], [19]. Second, psychological stress, like any form of threat, helps prepare individuals for adaptive courses of action, which might include physical attractiveness ideals [20]. Psychological stress signals a threat to the individual and should lead the individual to show a preference for more mature physical characteristics that would be beneficial during periods of threat [12].

In short, to the extent that heavier body sizes are perceived as more physically mature [17], it seems plausible that individuals experiencing psychological stress may experience a shift in their body size ideals. In order to test this hypothesis, we examined the effects of acute stress on men's body size preferences. By utilising a standardised stress test and by controlling for subjective perceptions of hunger, we were able to investigate the direct effects of stress on body size preferences. Based on previous work [16], [18], we predicted that men would show a preference for a larger female body size when experiencing psychological stress.

## Methods

### Ethical Statement

The ethics committee at the Department of Psychology, University of Westminster, specifically approved this study. All participants provided written informed consent.

### Participants

Participants were 81 heterosexual male undergraduates assigned to one of two groups: stress (*n* = 41) and control (*n* = 40), respectively. Because observer ethnicity may impact upon body size judgements [21], only British White participant were invited to take part in the present study. Participants in the former group ranged in age from 18 to 40 years (*M* = 21.73, *SD* = 3.67) and in body mass index (BMI) from 17.15 to 31.64 kg/m^2^ (*M* = 21.71, *SD* = 3.47). Participants in the latter group ranged in age from 18 to 42 years (*M* = 22.15, *SD* = 4.05) and in BMI from 16.53 to 27.76 kg/m^2^ (*M* = 21.37, *SD* = 3.70).

### Design and Procedure

Participants were recruited opportunistically by two research assistants from various campus locations. Upon arrival at the laboratory, participants provided informed consent and were randomly assigned to one of the two conditions. Participants in the stress condition took part in the Trier Social Stress Test (TSST) [22], a 15-minute laboratory stressor that has been reliably shown to increase levels of acute psychological stress (e.g., as measured by free cortisol levels) [22]–[23]. As part of the TSST, participants were given a 10-minute preparatory period, after which they were taken to a room in which four individuals (mixed genders) were already seated at a table and in which a video camera and tape recorder were installed. The participants stood at a microphone in front of the four individuals and took the role of a job applicant invited for a personal interview with a company's selection committee. Participants had to introduce themselves to the committee in a free speech of 5 minutes and attempt to convince the committee that they were suitable for the post. Participants were told that the committee was trained to monitor non-verbal behaviour and the voice and video analyses would be conducted. After 5 minutes, the selection committee asked the participants to serially subtract the number 13 from 1,022 as fast and accurately as possible. Standard responses were followed where participants ended their speech before the 5-minute duration or failed in the subtraction task [22].

Twenty minutes after the TSST, participants in the stress condition were taken to a separate room, where they completed the measures described below along with additional scales included to mask the study's aims. This delay is known to coincide with the onset of peak cortisol response following an acute psychological stressor [23]. By contrast, participants in the control group were taken immediately to a room where they waited quietly for the same length of time as the TSST stress-induction procedure before they completed the measures described below. Once the measures had been completed, participants had their body mass (kg) and height (cm) directly measured to the nearest 0.5 kg and 0.5 cm, without shoes and in light clothing, using a standard tape measure and weighing scale. All testing sessions took place between 3.00pm and 5.00pm in order to control for diurnal variations in cortisol secretion.

### Materials

#### Body size preferences

Participants completed an adapted version of the Photographic Figure Rating Scale [24], a figural rating scale that consists of 10 photographic and standardized images of women in front view. The women depicted in the PFRS represent the full range of established BMI categories, from emaciated to obese. Participants were first asked to rate each of the 10 images for physical attractiveness on a 9-point Likert-type scale (1 = *Very unattractive*, 9 = *Very attractive*). As in previous work [25], they were then asked to additionally rate the figure that they found most physically attractive (‘ideal’), the largest figure they found physically attractive (‘largest’), and the thinnest figure they found physically attractive (‘thinnest’). Responses on the latter three items were made on a 10-point scale, with 1 representing the figure with the lowest BMI and 10 representing the figure with the highest BMI. ‘Largest’ and ‘thinnest’ ratings were used to calculate an ‘attractiveness range’ (smallest figure that participants found attractive subtracted from the largest figure). Previous work has shown that scores derived from the PFRS have good patterns of validity and good test-retest reliability after a three-week interval [24], [26].

#### Appetite sensation

We assessed participants' subjective appetite sensations using the Appetite Sensation Assessment [27]. Participants were presented with 100 mm lines with words anchored at each end, describing extremes of hunger (*I am not hungry at all* versus *I have never been more hungry*) and satiety (*I am completely empty* versus *I cannot eat another bite*), fullness (*Not at all full* versus *Totally full*), and prospective food consumption (*Nothing at all* versus *A lot*). Participants were asked to mark across the line at the position on the scales corresponding to their feelings and quantification of the measurement was done by measuring the distance from the left end of the line to the mark. For the present purposes, an overall score of satiety was computed as the mean of all four responses, following reverse-scoring of appropriate items. Although based on self-reports, this method of appetite sensation measurement shows good retest-retest reliability, excellent reproducibility, and good validity [27]–[28].

## Results

### Sample Characteristics

Preliminary analyses using independent samples *t*-tests showed that there were no significant between-group differences in mean age, *t*(79) = 0.49, *p* = .627, *d* = 0.11, and mean BMI, *t*(79) = 0.43, *p* = .670, *d* = 0.10. There were also no significant between-group difference on the measure of appetite sensation, *t*(79) = 0.55, *p* = .585, *d* = 0.12. These preliminary analyses suggest that our randomisation procedure was successful. We also computed bivariate correlations between each of the dependent variables (ideal, largest, thinnest, and the attractiveness range, respectively) and participant age, BMI, and appetite sensation. Results showed that, with the exception of the correlation between largest and thinness ratings (*r* = .15, *p* = .193), the dependent variables were significantly and moderately correlated (*r*s = .34–.51, *p*<.001). The results also showed that the only significant correlation between a dependent variable and remaining factors was between ratings of the largest figure perceived as attractive and satiety (*r* = −.24, *p* = .034), which is consistent with previous work [3], [10]. All other correlations did not reach significance (*r*s = .05–.20, *p*s>.070).

### Body Size Ratings

Descriptive statistics (*Ms* and *SD*s) for ratings of each of the ten body size figures as a function of experimental group are reported in Table 1. In order to examine whether there were statistically significant between-group differences on these ratings, we calculated a multivariate analysis of covariance (MANCOVA), with body size ratings as the dependent variable, experimental condition as the independent variable, and participant age, BMI, and appetite sensation as covariates. Results showed a significant omnibus effect of experimental condition, *F*(10, 67) = 5.18, *p*<.001, with a moderate effect size (η_p_^2^ = .44). Covariate age, BMI, and appetite sensation did not reach significance in this analysis (*F*s = 0.34–1.23, *p*>.292, η_p_^2^<.16). Inspection of the ANCOVA results showed that there were no significant between-group differences in ratings of emaciated and underweight figures on the PFRS (*F*s = 0.04–1.42, *p*>.237, η_p_^2^<.02). On the other hand, participants in the stress condition provided significantly higher ratings than control participants for normal weight figures (*F*s = 14.31–17.26, *p*<.001, η_p_^2^ = .16–.19), overweight figures (*F*s = 15.45–15.97, *p*<.001, η_p_^2^ = .17), and one obese figure, *F*(1, 79) = 5.86, *p* = .018, η_p_^2^ = .07. There was no significant between-group difference on ratings of the figure with the highest BMI, *F*(1, 76) = 0.90, *p* = .346, η_p_^2^ = .01.

**Table 1:** Descriptive statistics of body size ratings as a function of experimental group.

Item		Control group (*n* = 40)		Stress group (*n* = 41)	
		*M*	*SD*	*M*	*SD*
Figure ratings^a^	Fig. 1	1.20	0.85	1.12	0.64
	Fig. 2	1.48	1.48	1.34	1.06
	Fig. 3	3.68	1.91	3.51	2.25
	Fig. 4	6.48	1.87	5.95	2.14
	Fig. 5	5.58	1.91	7.10	1.66
	Fig. 6	3.83	1.30	5.15	1.53
	Fig. 7	3.03	1.40	4.39	1.67
	Fig. 8	1.73	0.85	2.78	1.41
	Fig. 9	1.25	0.67	1.78	1.15
	Fig. 10	1.08	0.35	1.20	0.68
Most physically attractive (ideal)		3.90	0.55	4.44	0.67
Largest attractive figure		6.25	1.10	7.17	1.50
Thinnest attractive figure		3.28	0.72	3.34	0.66
Attractiveness range		2.98	1.35	3.83	1.45

Note:

a Figures 1 and 2 represent emaciated figures, 3 and 4 underweight figures, 5 and 6 normal weight figures, 7 and 8 overweight figures, and 9 and 10 obese figures.

We next examined ratings of the figure perceived as the most physically attractive (ideal), the largest and thinnest figures rated as attractive, and the attractiveness range (descriptive statistics are reported in Table 1). To do so, we computed analyses of covariance (ANCOVAs) with each of the aforementioned ratings as dependent variables, experimental condition as the independent variables, and participant age, BMI, and appetite sensation as covariates. Results showed that participants in the stress group rated a significantly larger figure as their ideal compared to the control group, *F*(1, 80) = 14.45, *p*<.001, η_p_^2^ = .16. The stress group also had a significantly wider attractiveness range than the control group, *F*(1, 80) = 6.63, *p* = .012, η_p_^2^ = .08. The latter effect appeared to be driven by the fact that the stress group rated a significantly heavier body size as the largest figure they considered attractive, *F*(1, 80) = 8.84, *p* = .004, η_p_^2^ = .10. By contrast, there was no significant between-group difference on ratings of the thinnest figure perceived as most attractive, *F*(1, 80) = 0.17, *p* = .683, η_p_^2^<.01. Covariate age, BMI, and appetite sensation did not have significant effects on between-group differences in ratings of the ideal, thinnest, or largest figures, or on the attractiveness range (*F*s = 0.21–1.01, *p*>.318, η_p_^2^<.02).

## Discussion

In the present study, we examined the impact of psychological stress on men's body size preferences using an experimental design. By comparing the preferences of an experimental group with a matched control group, we were able to focus on the specific effects of stress, while discounting possible confounding effects of age, BMI, and hunger. As expected, and consistent with the Environmental Security Hypothesis [15]–[16], [18], we found that the experience of stress shifted men's body size preferences, such that heavier female body sizes were rated more positively. Broadly speaking, the present results are consistent with the suggestion that individuals are more likely to idealise mature morphological traits, including a heavier body size, when they experience environmental threat, whether from economic [10], proprioceptive [3], [10]–[12], or social sources [18].

More specifically, the results of the present study showed that participants experiencing psychological stress selected a significantly heavier female body size as maximally attractive compared to the control group. Although the shift in preferences may appear small from a practical point-of-view, it should be noted that the effect size of the uncovered difference was moderate by conventional standards [29]. Additionally, our results showed that men who were stressed rated female body sizes at higher BMI categories as more attractive than their control group counterparts. That is, men in the experimental condition rated women of normal weight, overweight, and, partially at least, obese BMI categories as more attractive than the control group. These results are consistent with previous experimental work indicating that the experience of stress leads participants to prefer more mature physical characteristics [18], but extends earlier studies in showing that the stress also impacts on body size judgements.

Finally, the present results also showed that men in the experimental group idealised a wider range of female body sizes as being physically attractive compared to the control group. As before, the effect size of this between-group difference was moderate by Cohen's [29] standards. It was notable that this difference was driven by the shift in the experimental group's upper limit of attractive female bodies. That is, while there was no significant difference in the lower end of the range, the experimental group appear to have shifted the maximum cut-off for attractive bodies at higher BMIs, which resulted in their wider attractiveness range. This has some similarities with the female attractiveness preferences of male and female Zulus living in rural South Africa [4], who also showed a tolerance of a heavier body relative to British observers, which may be linked to their more stressful environment.

Taken together, the present results provide support for the suggestion that human attractiveness judgements are sensitive to variations in local ecologies and may reflect adaptive strategies for dealing with experiences of threat [4], [6], [30]. That is, human mate choice preferences are likely context-specific and recalibrate as local conditions and experiences change, the end result being mate preferences that remain adaptive regardless of the environmental landscape [4], [31]. The experience of stress may lead to a general preference for more mature physical traits in a potential partner because such traits are associated with improved ability to handle environmental stress [32]. More broadly, the present results may also help explain reported cross-cultural differences in ideal body size: in contexts marked by prolonged stress as a result of resource deprivation, individuals may idealise larger body sizes because such body types are associated with better ability to handle environmental threat [33].

Our results should be considered in the light of a number of limitations of our design. First, it is possible that having participants in the control group sit quietly without distractions impacted their levels of boredom, which in turn affected their body size judgments. It will, therefore, be important to replicate our findings using attention conditions that control for the task demands of the speech and math tasks, but that do not activate stress response systems [34]. Second, although the TSST is known to induce acute psychological stress and increase levels of cortisol, our design could be improved through more comprehensive measures of the stress variable. For example, measuring stress-induced cortisol, a glucocorticoid that is known to be related to cognitive functions [35] would allow for a more direct test of the associations between stress and body size preferences. At present, then, it cannot be fully established whether our stress procedure reliably activated stress response or sympathetic nervous systems (e.g., heart rate and blood pressure) in the intended manner. Future work could also manipulate when the body size judgements are collected: if stress-induced cortisol is indeed the mechanism that affects body size judgements, then collecting data on these judgements in the first three minutes post-TSST should produce no differential effects compared to collecting them 20 minutes post-TSST [36].

Second, it is possible that, in the present study, the experience of stress impacted on state self-esteem, empathy, or related constructs, which in turn may have impacted on body size perceptions. As such, in future work it may be necessary to control for these factors when examining the impact of stress on body size ideals. Third, because the PFRS currently only allows for the assessment of female body size judgements, we were not able to examine the effect of stress on women's judgements of male bodies. The available evidence from naturalistic designs would seem to point to a similar shift in preferences as a function of environmental threat [37]. Conversely, there is also evidence that hunger salience has differential effects on the preferences of women and men. Specifically, hunger state does not appear to alter female preferences for physical indicators of maturity (e.g., a heavier body size) to the same extent as it does for non-physical characteristics (e.g., more mature personalities) [17]. As such, future experimental research would do well to examine the effects of acute stress on female preferences for male body size. Finally, our reliance on a student sample means that our findings should only be generalised to the wider population with caution.

These limitations notwithstanding, the present results provide the first experimental evidence that the experience of psychological stress shapes men's judgements of female body size. Men experiencing stress not only perceive a heavier female body size as maximally attractive, but also more positively perceive heavier female body sizes and have a wider range of body sizes considered physically attractive. Although our work was focused on psychological stress, accumulating evidence suggests that different forms of stress (e.g., physiological, economic, social) have similar effects on physical attractiveness preferences [3], [16]–[18], [38]. These results underline the malleability of physical attractiveness judgements and have important implications for scholarly understanding of reported cross-cultural differences in body size ideals. Further research may also help to better explain reported within-cultural differences in physical attractiveness ideals [39], particularly if it can be established that chronic stress impacts upon such ideals.
